# A COVID-19 Airway Management Innovation with Pragmatic Efficacy Evaluation: The Patient Particle Containment Chamber

**DOI:** 10.1007/s10439-020-02599-6

**Published:** 2020-08-27

**Authors:** Lauren M. Maloney, Ariel H. Yang, Rudolph A. Princi, Alexander J. Eichert, Daniella R. Hébert, Taelyn V. Kupec, Alexander E. Mertz, Roman Vasyltsiv, Thea M. Vijaya Kumar, Griffin J. Walker, Edder J. Peralta, Jason L. Hoffman, Wei Yin, Christopher R. Page

**Affiliations:** 1 Department of Emergency Medicine, Stony Brook University Hospital, Stony Brook, NY USA; 2 Department of Biomedical Engineering, Stony Brook University, Stony Brook, NY USA; 3 Renaissance School of Medicine at Stony Brook University, Stony Brook, NY USA; 4 Department of Mechanical Engineering, Stony Brook University, Stony Brook, NY USA; 5 School of Health Technology and Management, Stony Brook University, Stony Brook, NY USA; 6 Department of Technology and Society, Stony Brook University, Stony Brook, NY USA; 7 Department of Psychology, Stony Brook University, Stony Brook, NY USA; 8 Department of Physics and Astronomy, Stony Brook University, Stony Brook, NY USA; 9 Department of Anesthesiology, Stony Brook University Hospital, Stony Brook, NY USA

**Keywords:** COVID-19, Aerosol, Intubation, Airway, Personal protective equipment, Emergency medical services

## Abstract

The unique resource constraints, urgency, and virulence of the coronavirus disease 2019 pandemic has sparked immense innovation in the development of barrier devices to protect healthcare providers from infectious airborne particles generated by patients during airway management interventions. Of the existing devices, all have shortcomings which render them ineffective and impractical in out-of-hospital environments. Therefore, we propose a new design for such a device, along with a pragmatic evaluation of its efficacy. Must-have criteria for the device included: reduction of aerosol transmission by at least 90% as measured by pragmatic testing; construction from readily available, inexpensive materials; easy to clean; and compatibility with common EMS stretchers. The Patient Particle Containment Chamber (PPCC) consists of a standard shower liner draped over a modified octagonal PVC pipe frame and secured with binder clips. 3D printed sleeve portals were used to secure plastic sleeves to the shower liner wall. A weighted tube sealed the exterior base of the chamber with the contours of the patient’s body and stretcher. Upon testing, the PPCC contained 99% of spray-paint particles sprayed over a 90s period. Overall, the PPCC provides a compact, affordable option that can be used in both the in-hospital and out-of-hospital environments.

**Electronic supplementary material:**

The online version of this article (10.1007/s10439-020-02599-6) contains supplementary material, which is available to authorized users.

## Introduction

Recent evidence has shown that severe acute respiratory syndrome corona virus 2 (SARS-CoV-2) may remain infectious as an aerosol for at least 3 h.^29^ Although the exact mode of SARS-CoV-2 transmission remains is still under investigation, it is likely that airborne transmission *via* aerosols and droplets is a significant factor in human-to-human spread.^31^ Given that the coronavirus disease 2019 (COVID-19) caused by SARS-CoV-2 is predominantly a pulmonary disease,^1^,^22^ it follows that moderately to severely ill patients will often require some form of airway intervention.^6^,^23^ Secretions coming from the upper and lower airways have been found to contain high viral loads.^24^,^30^ Therefore, aerosol-generating airway interventions such as ventilations with a bag-valve mask, endotracheal intubation and extubation, as well as tracheal suctioning place healthcare providers at increased risk for viral exposure.^7^,^11^,^26^ This risk of exposure is further complicated by a widespread concern about the availability of and access to appropriate personal protective equipment (PPE).^3^,^25^ Therefore, innovative approaches to protect healthcare providers from aerosols and droplets generated during airway interventions is an integral component of limiting the spread of the SARS-CoV-2 virus.

In late March 2020, Taiwanese anesthesiologist Dr. Lai Hisen-Yung began to develop the Aerosol Box: a transparent acrylic box to be placed over a patient, with an open side facing the chest and two holes through which a healthcare provider can insert their hands in order to perform airway interventions.^13^,^15^,^27^ A simulated patient cough using compressed oxygen to explode a dye-containing balloon suggested that without the Aerosol Box, dye droplets were visible on the healthcare provider’s face, torso, and floor, compared to just the healthcare provider’s hands and forearms with the Aerosol Box in place.^8^ This barrier device was rapidly adopted by hospitals internationally even without adequate evidence about its efficacy. This was due in large part to dissemination *via* social media sharing.^10^,^27^

However, difficulties with the Aerosol Box were soon realized. It was found to be restrictive, can create tears in healthcare providers’ PPE, interferes with the use of a video laryngoscope, and is subject to glare from overhead lighting. In addition, it has a very limited ability to adapt to healthcare provider height or to patient body habitus.^8^,^10^,^18^,^28^ Additional obstacles include managing the device’s weight and bulkiness during transport, securing the device to the bed especially if the head is elevated, and proper decontamination and storage procedures.^10^ Although multiple iterations of the Aerosol Box have been devised addressing some of these concerns, very limited overall data exists to quantify its ability to protect staff present in the patient’s room.

The use of barrier devices to limit airborne transmission during airway interventions so far has been limited to in-hospital settings. Many of the critiques of the Aerosol Box would likely be amplified in an out-of-hospital environment. This is due to the constraints of ambulance stretcher dimensions, portability, and storage. Additionally, the patient care compartment of an ambulance is generally much smaller than a critical care procedure room in an Emergency Department, operating room, or inpatient hospital room. Performing airway interventions for patients suspected of having coronavirus disease 2019 (COVID-19) in an ambulance in accordance with current consensus recommendations^7^,^11^ is further complicated by patient compartments rarely being equipped to induce negative-pressure, and the lack of national standards for ambulance ventilation systems in the United States.^20^

Our primary objective was to create a barrier device with the ability to reduce transmission of airborne particles generated during airway interventions that is portable and could be assembled from commonly available components that are unlikely to be in short supply during the pandemic. Critical design criteria for the device included the reduction of transmission of airborne particles by at least 90% as measured by pragmatic testing; construction using materials outside of the traditional hospital supply chain which can be readily obtained on a limited budget and timeline; any reusable components are easy to clean; and dimensions allowing for use within the limited space of commonly used EMS stretchers.

## Materials and Methods

### Setting

This device was created and pragmatically tested at Stony Brook University Hospital, in Stony Brook, NY, USA. Stony Brook University Hospital is a suburban, academic tertiary care center, located on a shared campus with Stony Brook University.

### Patient Particle Containment Chamber (PPCC)

**Figure 1:**
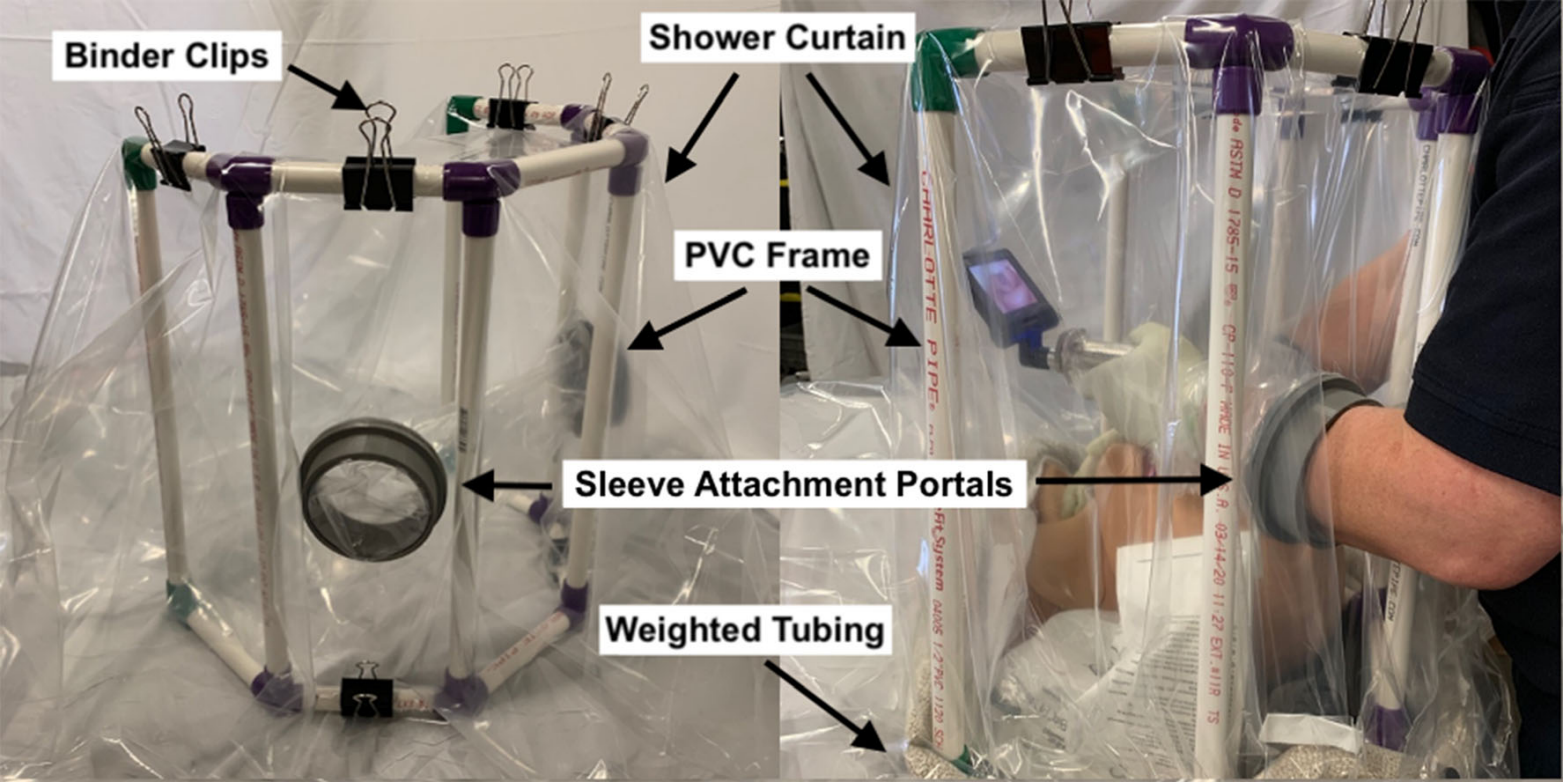
The Patient Particle Containment Chamber with labeled components.

Supplementary Material A describes the assembly process in detail. Briefly, as illustrated in Fig. 1, the PPCC frame was constructed from ½ in. PVC pipes and PVC fittings (Fig. 1). A clear shower liner was draped over the frame and secured in place using 2 in. binder clips. 3D printed portals were used to mount plastic sleeves onto the shower liner wall. Seven-inch diameter 3mil poly tubing was used for the plastic sleeve material. A weighted tube was draped along the exterior base of the chamber and secured in place using double sided tape.

### Contamination Evident Chamber

A cube-shaped support structure was assembled using ½ in. ×2 in. ×48 in. poplar boards. The cube was lined using 48 in. wide easel paper, creating the Contamination Evident Chamber.

An investigator placed a gowned arm into the Containment Evident Chamber *via* a small hole cut in the easel paper and sprayed a can of black spray paint within the chamber in all directions for 90 s. Paint was visually seen on all six surfaces after the 90 s. The paint was allowed to dry. This served as the control to compare to the performance of the PPCC to.

The paper of the chamber was changed, and the PPCC was placed within it. An identical can of black spray paint was placed inside the PPCC and was sprayed in all directions for 90 s by the same investigator as before. The paint was allowed to dry.

### Measurements

After being cut into 8.5 × 11” sections, the easel paper was scanned into a digital image. ImageJ software (NIH, Bethesda, MD) was then used to determine the surface area covered by black paint. The total area of paint coverage was measured in terms of pixels and then converted to units of square inches using the appropriate conversion factors.

## Results

Without the PPCC in place, the total surface area of the Contamination Evident Chamber covered by black paint was 2312 square inches. With the PPCC in place, less than 1 square inch of surface area outside of the PPCC was covered by black paint, and it was limited to only the floor surface of the Contamination Evident Chamber. The area of the Contamination Evident Chamber underneath the PPCC, as well as the inner surfaces of the walls of the PPCC, were completely covered in black paint. The exact margins of the PPCC were difficult to ascertain, as the folds of the shower liner material draped onto the floor in an irregular shape, as intended, and often in several layers. By geometry, the PPCC frame covers 232 square inches of the floor surface of the Contamination Evident Chamber. Conservatively factoring in a possible 10% additional surface area that may be covered by the layers of shower liner, approximately 256 square inches of the floor surface of the Contamination Evident Chamber is covered by the PPCC. Reducing this area from the total surface area of the Contamination Evident Chamber covered in black paint without the PPCC in place infers that 2,056 square inches of paint covered area was outside the area encapsulated by the PPCC. Therefore, the PPCC reduced the total surface area of black paint in the Contamination Evident Chamber by more than 99%.

The total cost per PPCC was $63.70. This figure includes the cost of shipping for products purchased online, as well as the local sales tax of 8.6%. The cost of the re-usable components was $52.18. The cost for the single-use disposable components was $11.52. Of note, this does not include the cost of labor or the 3D printing materials.

## Discussion

In response to the need for innovative ways to safely perform airway interventions on patients with COVID-19 in the setting of limited availability of personal protective equipment, we designed a Patient Particle Containment Chamber. The device was made from readily available materials that are outside the traditional hospital supply chain. Total materials cost was approximately $60. In comparison, commercially-made solutions available for online purchase range in cost from $60-270 (excluding tax and shipping costs), and are dependent upon specific companies’ material production and their supply availability.^16^,^17^,^19^,^27^ Pragmatic testing of the chamber using a can of black spray paint suggests that it is at least 99% effective in reducing the transmission of small airborne particles.

The creation and subsequent almost immediate world-wide adoption of the Aerosol Chamber was a clear demonstation of the power of idea dissemination through social media. However, the wildfire spread of the device was followed by abrupt critiques about its functionality, illustrating the cautionary tale proposed by Duggan *et al*., for which they proposed the term “MacGyver bias.”^12^ The MacGyver bias is “the inherent attraction of our own personal improvised devices. This leads to a tendency to hold them in high regard despite the relative absence of evidence for their efficacy.”^12^ While necessity, especially when faced with a highly contagious pandemic, may very well be the mother of all invention, it is crucial that thought is given to the determination of whether or not the device does indeed do what is intended. Homemade devices may come from a lack of existence of or lack of access to a suitable commercially-available device. In such cases, it is important to ponder how and if an innovative solution would have undergone iterative changes in order to meet regulatory safety standards. Given the speed with which COVID-19 traversed the globe, traditional innovation timelines have been markedly shortened. Adding to this, has been disruption of hospital supply chains, leading to rapid depletion of PPE. Furthermore, well-intentioned clinicians creating these temporary solutions may lack a background in the medical device innovation process. This may increase the possibility of oversights in areas including safety mechanisms, material selection, design tolerance, and product testing.

While the pragmatic testing of our proposed chamber in reduction of airborne particle transmission would certainly not conform to NIOSH standards, we believe using a can of black spray paint is a reasonable demonstration of efficacy. Reports suggest that airborne particles generated by standard spray paint cans are of a size comparable to that which is necessary to be defined as an infectious aerosol particle.^14^,^21^ Furthermore, this technique for testing can be easily replicated by others if they would like to evaluate the efficacy of their own devices for a cost of less than $5.

Quantitative image analysis suggested that the device was able to prevent almost all simulated aerosolized particles from leaving the device. Existing published descriptions of attempts to evaluate how useful barrier devices are at containing airborne particles have been limited to a qualitative description of fluorescent dye splatter patterns^8^ and a video anlysis of white vapor clouds flowing out of the Aerosol Box.^28^ A quantitative approach was described in the evaluation of the utility of a constant flow canopy, a flexible polyethylene canopy that is placed over the head and upper thorax of patient while in a hospital bed which contains a fan filtering unit and HEPA filter.^2^ However, this device is not designed to allow any sort of airway intervention, but rather could limit the ambient release of aerosols from patients undergoing noninvasive ventilation support. As smoke was piped under the canopy, the face velocity and direction of the smoke movement was determined, while photometry was used to evaluate the integrity of the HEPA filter.^2^

A single simulation-based study has been published evaluating the impact of two generations of Aerosol Boxes on simulated endotracheal intubation by anesthesiologists; however, it was not structured to evaluate the efficacy of such devices on reducing airborne particle exposure to clinicians.^5^ It was found that with the Aerosol Box in place, anesthesiologists took longer to intubate, had fewer successful first attempts, increased provider cognitive load, and increased provider discomfort during the procedure. Most concerningly, was the number of breaches in PPE that resulted from the gown getting stuck or torn in the arm holes of the Aerosol Box.^5^

Having already undergone several iterations, we have shown that our device successfully addresses many of these issues. The Patient Particle Containment Chamber provides plenty of space for unhindered airway interventions such as using a handheld video laryngoscope or airway adjuncts. When not in use, it can be collapsed and stored easily. Reusable components are easy to clean and disinfect. Additionally, it is unlikely that gowns will be torn given the mobile nature of the sleeve attachment portals. Although access to a 3D printer is necessary to create the sleeve attachment portals, news reports suggest access to this equipment is more universal than ever before, particularly through local libraries and high schools.^4^,^9^

Future iterations of the Patient Particle Containment Chamber will need to address several design elements. First, in order to consider leaving this device in place for extended periods of time to allow for longer airway interventions such as a nebulized medication, hourly tracheal suctioning, or extubation, determination of a safe time period that the device can be used before ambient oxygen is depleted or carbon dioxide accumulates must be made. A system similar to the fan filtering unit and HEPA filter described by Adir *et al*. could be considered in order to better control air and particle flow.^2^ This may also help mitigate dangerous diffusion of aerosols contained within the chamber into the surrounding air once the device is removed. Alternatively, we have created a miniature version of the sleeve attachment portal which would allow for ventilator tubing to pass through the chamber. These could be configured to allow for a bag-valve mask to be attached to the chamber to introduce supplemental oxygen, as well as adapted to allow for any outflow to be filtered. Several components of the chamber could be prepared in advance in order to shorten the assembly time, and it may be useful to include elastic cord within the top and bottom partial octagons of the frame to allow for faster frame assembly. Availability of completely prefabricated fittings may help to remove the need to glue pieces together. Specific to its out-of-hospital use, the chamber frame fits well on the top of the most common EMS stretchers, though additional stability could be added by using a clamp to hold the bottom of the frame to the stretcher mattress. This could reduce the potential for the device to topple off of the patient during transport or when the head of the stretcher is raised. Finally, the chamber needs to undergo formal compliance testing, similar to what is done for laboratory ventilator hoods, before its efficacy can be reported with precision.

This study was limited to a single attempt made to pragmatically quantify the amount of black spray paint that was apparent on the inner surfaces of a 4-foot cube with and without the chamber in place. The data that we are able to report is limited due to a lack of ability to determine the exact edge of the enclosure. The irregular, multilayered way the shower liner was wrapped around the weighted tube did not allow for clearly defined boundaries. The initial iteration used a 6 foot long dishwasher tube filled with water and occluded at each end as the weighted tube; while it appeared to work, later iterations using pellet-filled 2 inch wide 2 Mil poly tubing instead offered more compliance across surface contours and easier use.

The current COVID-19 pandemic has demonstrated the lack of devices available to ensure provider safety during airway interventions. The device described provides a promising solution to help protect both hospital and out-of-hospital providers during airway interventions. In addition, the design of the Patient Particle Containment Chamber prioritizes the use of readily accessible and economically affordable parts in order to avoid contributing to a shortage of hospital equipment during surges of patients. While current constraints prevent the device from being formally tested, initial pragmatic evaluation using airborne particle simulation and imaging analysis yields highly promising results. Although the device may not solve all current concerns about healthcare provider safety against respiratory viral infections, the PPCC provides an additional, more versatile option to help limit transmission of SARS-CoV-2 during high risk airway interventions.

## Electronic supplementary material

Below is the link to the electronic supplementary material.

Supplementary material 1 (DOC 2373 kb)

Supplementary material 2 (DOC 308 kb)

Supplementary material 3 (ZIP 31 kb)

## Abbreviations

COVID-19: Coronavirus disease 2019
EMS: Emergency medical services
HEPA: High efficiency particulate air
NIOSH: National Institute of Occupational Safety and Health
PPCC: Patient Particle Containment Chamber
PPE: Personal protective equipment
PVC: Polyvinyl chloride
SARS-CoV-2: Severe acute respiratory syndrome corona virus 2
