# Supplementary material for: A COVID-19 Airway Management Innovation with Pragmatic Efficacy Evaluation: The Patient Particle Containment Chamber
Source: Ann Biomed Eng. 2020 Aug 27;48(10):2371–6. doi: 10.1007/s10439-020-02599-6 (PMC7453071; doi:10.1007/s10439-020-02599-6)
Supplement: Supplementary file 1 — Supplementary material 1 (DOC 2373 kb) [file 10439_2020_2599_MOESM1_ESM.docx]

**Supplementary Material A: Patient Particle Containment Chamber Assembly Instructions**

**Equipment**

- PVC pipe cutter
- Ruler
- Thin permanent marker
- Scalpel or box cutter
- PVC cement

**Materials**

- PVC pipe, 14 ft of 0.5 inch inner diameter
- (2) 2 inch wide 2 Mil poly tubing, 8 ft
- Poly pellets, 2 lbs
- (4) ½ inch 90 degree PVC fitting
- (8) ½ inch 45 degree PVC fitting
- (8) ½ inch external PVC flat end cap
- Standard shower liner, clear, 72 in. x 72 in.
- (6) 2 inch binder clips
- (2) sleeve attachment portals (Supplementary Material B and C)


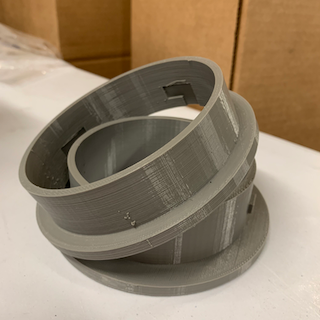


- (2) 7 inch wide 3 Mil poly tubing, 2.5 ft
- Double-sided mounting tape, 2 ft of ¾ inch tape
- Pair of surgical gloves

**Preparation**

1. Cut PVC pipe into smaller sections: (10) 6” long; (6) 18” long.
2. To create the weighted tube, tie a knot at the end of one 8 ft section of 2 inch wide 2 Mil poly tubing, fill with 2 lbs of poly pellets, then tie a knot at the remaining open end.
3. Use PVC cement to attach a flat end cap to each 45 degree fitting. Let set overnight.


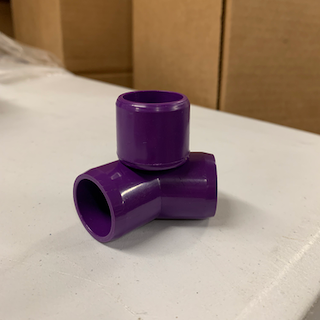


**Assembly Instructions:**

1. Assemble 2 partial octagons using the 7” PVC pipe sections and fittings.


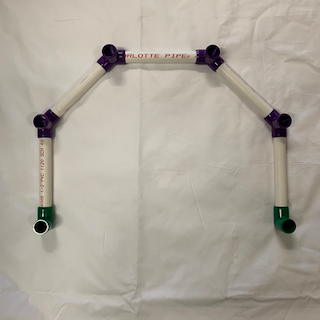


1. Use the 18” PVC pipe sections to attach the 2 partial octagons together, creating the frame.


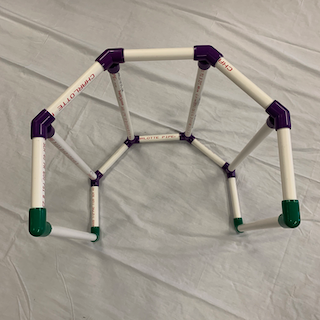


1. Drape shower liner over the frame, using binder clips to secure the liner to the frame


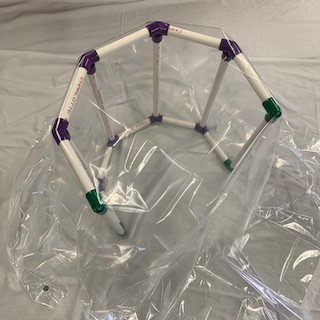

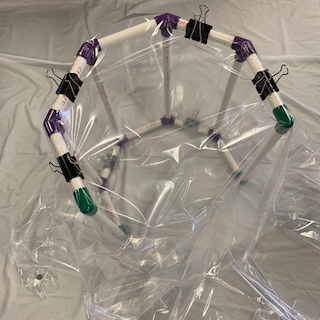

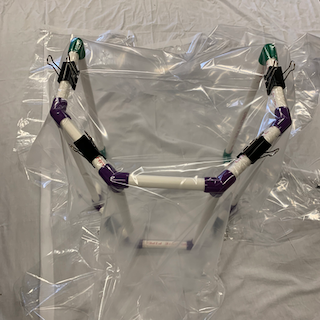


1. Use a scalpel or box cutter to cut holes in the shower liner for the sleeve attachment portals.
2. Use the sleeve attachment portals to attach the 7 inch wide poly tubing sections to the shower liner. Of note, ensure the part generated from the file named “inside_sleeve_attachment_portal” is inside the chamber. The shower liner is pulled taut against the inside sleeve attachment portal. The outside sleeve attachment portal is then placed over the inside sleeve attachment portal and rotated to lock it in place.


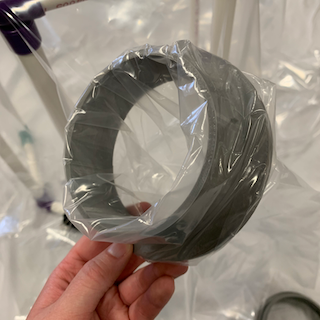

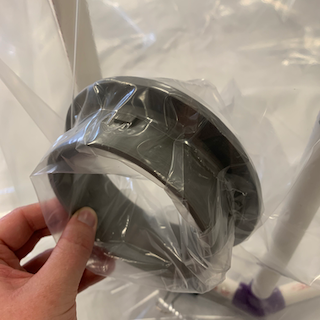

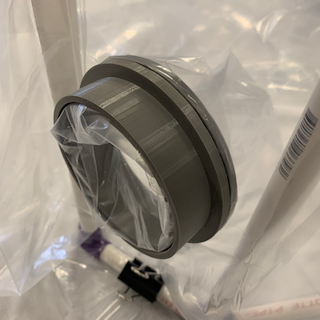


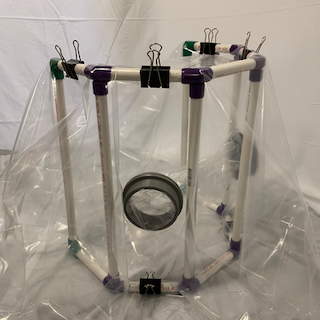


1. Use the remaining section of 2 inch wide poly tubing as a protective outer cover for the 2 inch wide poly pellet containing weighted tube.
2. Place Patient Particle Containment Chamber over patient. Airway intervention supplies and pair of sterile gloves should be placed on bed next to patient’s head, within the area covered by the chamber.
3. Drape weighted tube around the base of the chamber.


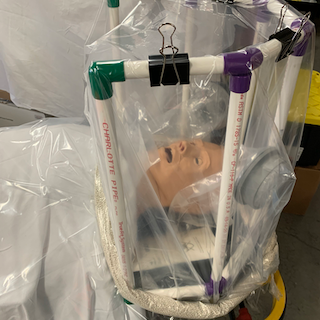


1. Use double sided tape to wrap and secure the shower liner around the weighted tube.
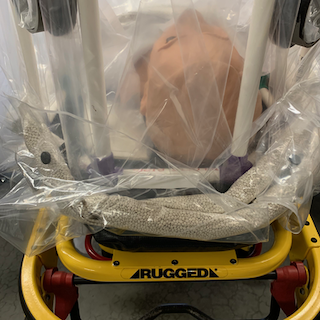

2. When ready to perform airway intervention, insert gloved hands through sleeve attachment portals. Poke a hole for each thumb near the end of the 7 in. wide 3 Mil poly tubing sleeves. Don the sterile gloves that are within the chamber, making sure the cuff of the gloves are over the poly tubing sleeves.


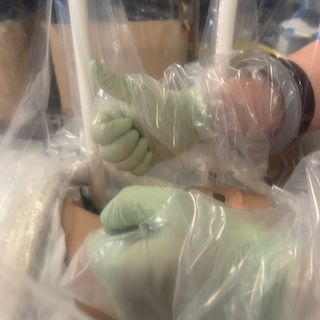

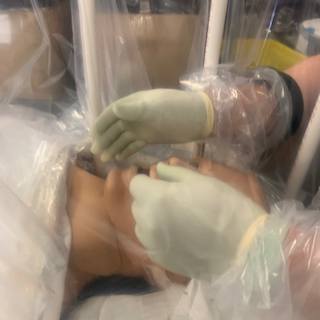


**Doffing Instructions:**

1. When the airway intervention is complete, remove the sterile gloves within the PPCC.
2. Remove hands from plastic sleeves.
3. Remove and discard inner pair of gloves.

**Disassembly Instructions:**

1. Free weighted tube from shower liner, and then discard outer protective layer of plastic.
2. Free shower liner from frame by removing binder clips.
3. Unfasten sleeve attachment portals.
4. Discard everything except the following: frame, sleeve attachment portals, binder clips, and weighted tube. The surface of these components should be disinfected according to standard institutional/agency cleaning practices for comparative patient care items.

*To make assembly at time of patient intervention faster, the partial octagon sections of the frame and Assembly Instruction Steps 4-6 could be done ahead of time.*
