# Supplementary material for: A COVID-19 Airway Management Innovation with Pragmatic Efficacy Evaluation: The Patient Particle Containment Chamber
Source: Ann Biomed Eng. 2020 Aug 27;48(10):2371–6. doi: 10.1007/s10439-020-02599-6 (PMC7453071; doi:10.1007/s10439-020-02599-6)
Supplement: Supplementary file 2 — Supplementary material 2 (DOC 308 kb) [file 10439_2020_2599_MOESM2_ESM.docx]

**Supplementary Material B: Sleeve Attachment Portal Schematics**

******

******
